# Differences in the Neural Substrate for Physical and Mental Quality of Life in Patients With Multiple Sclerosis

**DOI:** 10.1002/brb3.71050

**Published:** 2025-11-21

**Authors:** Juichi Fujimori, Michiko Nei, Shu Umezawa, Tatsuro Misu, Ichiro Nakashima

**Affiliations:** ^1^ Division of Neurology Tohoku Medical and Pharmaceutical University Sendai Japan; ^2^ Nursing Bureau Tohoku Medical and Pharmaceutical University Hospital Sendai Japan; ^3^ Department of Neurology Tohoku University Graduate School of Medicine Sendai Japan

**Keywords:** cortical thickness, demyelinating disease, mental health, neurodegenerative disease

## Abstract

**Introduction:**

Identifying patients with multiple sclerosis (PwMS) who primarily experience a gradual decline in mental rather than physical quality of life (QoL) is clinically significant, as QoL worsening may be underestimated. This study aims to compare the clinical characteristics and neural substrates of PwMS with predominantly reduced physical and mental QoL to distinguish between these patients.

**Methods:**

This study included 75 PwMS, of whom 56 had relapsing‐remitting multiple sclerosis (MS) and 19 had progressive MS. The Multiple Sclerosis Neuropsychological Questionnaire (MSNQ) and Medical Outcomes Study Short Form‐36 Health Survey were used to assess neuropsychological abnormalities and health‐related QoL.

**Results:**

Physical and mental QoL were not correlated. In a subset of PwMS, cluster analysis revealed that either physical or mental QoL was primarily affected. Patients with reduced physical QoL (*n* = 9) were generally older, more likely to have a progressive disease course, and had higher EDSS scores. Imaging revealed reduced whole‐brain and grey matter volumes, as well as bilateral cortical atrophy in the frontal lobes, specifically in the left rostral middle frontal cortex. In contrast, patients with reduced mental QoL (*n* = 19) had a relapsing‐remitting disease course and elevated MSNQ scores. These patients exhibited an increased lesion load, T1 white matter hypointensity volume, and bilateral cortical atrophy in the temporal lobes, specifically in the right insula and left superior temporal cortex.

**Conclusion:**

PwMS with predominantly reduced mental QoL exhibit distinct inflammatory changes that may contribute to disrupted connectivity and cortical atrophy in the lateral postcentral regions.

## Graphical abstract

The present study found no significant correlation between reduced physical and mental quality of life (QoL) in patients with multiple sclerosis (MS). Patients whose mental (green dots) and physical (blue dots) QoL was diminished exhibited decreased cortical thickness in the bilateral temporal and frontal lobes, respectively.

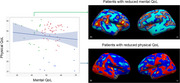

**Abbreviations**
3D: 3‐dimensional
DLPC: dorsolateral prefrontal cortex
DMT: disease‐modifying therapy
EDSS: Expanded Disability Status Scale
FLAIR: fluid‐attenuated inversion recovery
FoV: field of view
HRQoL: health‐related quality of life
IQR: interquartile range
MCS: mental component summary
MRI: magnetic resonance imaging
MS: multiple sclerosis
MSNQ: Multiple Sclerosis Neuropsychological Questionnaire
PCS: physical component summary
PMS: progressive multiple sclerosis
QoL: quality of life
RRMS: relapsing‐remitting MS
SD: standard deviation
SF‐36: Medical Outcomes Study Short Form‐36 Health Survey
TE: echo time
TI: inversion time
TR: repetition time
WM: white matter

## 1 Introduction

Multiple sclerosis (MS) is a demyelinating and neurodegenerative disease of the central nervous system that occasionally affects young adults (Haki et al., 2024; Thompson, Baranzini, Geurts, Hemmer, and Ciccarelli, 2018b). Neurodegeneration contributes to physical and cognitive disability, which negatively impacts everyday functions and quality of life (QoL) (Amato et al. 2019). Moreover, factors such as age, sex, socioeconomic and disability status, depression, and fatigue have been associated with health‐related QoL (HRQoL) in patients with MS (Berrigan et al. 2016). Consequently, patients with MS tend to report lower QoL than the general population.

QoL can be subdivided into physical and mental components. Patients with MS may experience a decline in both; however, each may be affected to different extents. For example, cognitive impairments impacting mental QoL are present in the early stages of the disease, even in individuals with low levels of physical disability, as measured using the Expanded Disability Score Scale (EDSS) (Amato et al. 2019). In contrast, higher EDSS scores consistently correlate with physical disability. No clear association between EDSS scores and mental QoL has been identified (Asadollahzadeh et al. 2024). These findings suggest that a subset of patients with MS may predominantly experience poor physical or mental QoL.

Identifying patients with MS with a predominant decline in mental QoL is essential for several reasons. Symptoms such as cognitive impairment, fatigue, depression, and pain are highly prevalent, affecting more than 50% of patients with MS (Heitmann et al. 2022). However, the effects of these symptoms on QoL can be more subtle and insidious than those of other symptoms typically associated with MS, such as visual or gait abnormalities, and are often overlooked by clinicians and patients in the early stages of the disease. Consequently, patients with MS primarily affected by mental QoL impairment may be under‐recognized, resulting in delayed or suboptimal treatment. Therefore, a holistic diagnostic approach is crucial for early identification and timely intervention. In addition, understanding the differences in the underlying causes of the impairment in physical and mental QoL at the neural level may improve patient outcomes.

To date, numerous studies have examined the association between cortical areas and physical or mental QoL independently. For instance, dysfunction of the mesocorticolimbic system, which includes the prefrontal cortex, striatum, and limbic system, has been linked to MS‐related depression, fatigue, and pain (Heitmann et al. 2022). In contrast, a recent study reported that chronological cortical thickness changes correlated with EDSS changes in patients with MS, primarily in the bilateral prefrontal, frontal, and temporoparietal regions (Tsagkas et al. 2020). These findings suggest that cortical areas associated with physical and mental QOL may overlap.

In this study, we first evaluated whether subsets predominantly affected by physical or mental impairments in their QoL can be defined within patients with MS. We used the Medical Outcomes Study Short Form‐36 Health Survey (SF‐36), which assesses QoL through the physical component summary (PCS) and mental component summary (MCS). Next, we investigated the clinical characteristics and neural substrates associated with these subsets. Based on Luria's model of cortical functioning, which conceptualizes the cerebral cortex as comprising two functional units (Luria 1973; Pena‐Casanova et al. 2024)—one for obtaining, processing, and storing information coming from the external environment (lateral postcentral regions of the neocortex on the convex surface of the hemispheres) and the other for programming, regulating, and verifying mental activity (precentral anterior areas of the hemispheres)—we hypothesized that patients with MS with decreased physical QoL may exhibit cortical atrophy in the precentral anterior region, whereas those with decreased mental QoL may be affected in the lateral postcentral region. We tested this hypothesis by comparing cortical thickness between these two subsets of patients.

## 2 Materials and Methods

### 2.1 Patients

We prospectively recruited 75 Japanese patients with MS who attended the Department of Neurology at Tohoku Medical and Pharmaceutical University Hospital, Sendai, Japan, in 2023. The inclusion criteria were: MS as defined by the 2017 revision of the McDonald criteria (Thompson et al. 2018a) and age between 20 and 70 years. Patients who were positive for anti‐aquaporin 4 antibodies and/or anti‐myelin oligodendrocyte glycoprotein antibodies either in serum or cerebrospinal fluid (determined using a cell‐based assay), and those with a history of psychiatric illness other than stable depressive symptoms, were excluded. Patients with missing data were also excluded. Physical disability was assessed using the EDSS (Zivadinov et al. 2025). Neuropsychological abnormalities were evaluated using the patient‐reported Multiple Sclerosis Neuropsychological Questionnaire (MSNQ) (Muryoi, Nei, Fujimori, and Nakashima 2025), comprising 15 questions that assess cognitive impairments on a scale from 0 (never) to 4 (frequently). The total MSNQ score was used as the dependent variable. The questionnaire captured deficits in processing speed, attention, memory, executive function, and behavior. Additionally, 72 out of the 75 patients with MS were evaluated for information processing speed using CogEval (Biogen Inc., Cambridge, USA), an iPad‐based screening tool validated against the Symbol Digit Modalities Test (Kalb et al. 2018; Rao et al. 2017). Data from the remaining three patients was not available. The study was conducted in accordance with the ethical standards outlined in the 1964 Declaration of Helsinki and its subsequent amendments and was approved by the Institutional Ethics Committee of Tohoku Medical and Pharmaceutical University (2023‐2‐002). Written informed consent was obtained from all the participants.

### 2.2 SF‐36

HRQoL was evaluated in all participants using the Japanese version of the SF‐36 (Fukuhara et al. 1998), which assesses 36 items regarding general well‐being. All 36 items have 3–5 possible answers scored on a scale of 1–5. The items are scored on eight subscales: physical functioning, physical role, bodily pain, general health, vitality, social functioning, emotional role, and mental health. According to the specific subscales being considered, either the PCS or the MCS can be calculated to characterize QoL. The PCS is derived from the physical functioning, physical role, bodily pain, general health, and vitality subscales. The MCS is derived from mental and general health, vitality, social functioning, and emotional role subscales (Fujio et al. 2017). Higher scores indicate a better QoL. Data was converted to norm‐based scoring, with a mean of 50±10. The references are based on a Japanese national survey conducted in 2007. As the structure of the SF‐36 questionnaire for Asian and Western countries differs, a three‐component model consisting of PCS, MCS, and social role component summary (RCS) has been developed, and its use is recommended in Japan. However, our previous study on patients with MS demonstrated that the findings using either the two‐component (PCS and MCS) or three‐component (PCS, MCS, and RCS) models were similar (Muryoi et al. 2025). Consequently, we defined the subsets of patients with MS according to the decline in physical or mental QoL using the two‐component model.

### 2.3 Hierarchical Cluster Analysis

A hierarchical cluster analysis using PCS and MCS was performed to classify patients with MS into several subgroups. Clustering is a multivariate technique that groups together observations that share similar values across multiple variables. We used Ward's clustering linkage method to combine clusters (Ward 1963). Thereafter, the silhouette value, Calinski‐Harabasz Index, Davies–Bouldin score, and Bootstrap Jaccard Index were applied to evaluate the cluster validity using the scikit‐learn library in Python version 3.12.0 (Pedregosa et al. 2011). The silhouette coefficient ranges between ‐1 and 1, with a higher coefficient corresponding to a model with more coherent clusters. A negative value indicates that the samples could have potentially been assigned to the wrong cluster (Belyadi and Haghighat 2021). The Calinski‐Harabasz Index is the ratio of the sum of between‐clusters dispersion and of within‐cluster dispersion for all clusters. The score is higher when clusters are dense and well separated (Caliński and Harabasz 1974). Regarding the Davies‐Bouldin score, the minimum score is zero, with lower values indicating better clustering. Bootstrap Jaccard Index analysis involves generating bootstrap replicates of the original dataset and performing the clustering algorithm on each replicate. The stability of each cluster was quantified by computing the mean Jaccard coefficient between the cluster in the original data and the most similar cluster identified in the bootstrap replicates (Hennig 2007). The resulting mean Jaccard Index ranges from 0 to 1.

### 2.4 Magnetic Resonance Imaging (MRI) Acquisition

All participants underwent MRI on the same whole‐body 1.5 Tesla MRI system (MAGNETOM Aera, Siemens, Germany). The MRI acquisition protocol involved a high‐resolution sagittal 3‐dimensional (3D) T1‐weighted magnetization‐prepared rapid gradient‐echo (MPRAGE) sequence (repetition time [TR]: 2730 ms; echo time [TE]: 3.3 ms; inversion time [TI]: 1000 ms; 176 slices; field of view [FoV]: 256 mm; and measured isotropic voxel size: 1 × 1 × 1 mm) and a sagittal 3D fluid‐attenuated inversion recovery (FLAIR) sequence (TR: 5000 ms; TE: 335 ms; TI: 1800 ms; 176 slices; FoV: 256 mm; and measured isotropic voxel size: 1 × 1 × 1 mm).

### 2.5 MRI Post‐Processing to Measure Global and Regional Brain Volumes

The 3D‐MPRAGE dataset served as the input data for the post‐processing pipeline. Regional and whole‐brain volumes were estimated using the automated FreeSurfer stream (version 7.1.0; https://surfer.nmr.mgh.harvard.edu/) as previously described (Fujimori, Fujihara, et al. 2020b; Fujimori et al. 2021; Fujimori, Uryu, et al., 2020; Nishizawa et al. 2022). Brain volume data for 63 segments were extracted from the automated segmentation results for each patient obtained from FreeSurfer and normalized to their head size using the total intracranial volume (TIV). These unitless values were used in further analyses.

To assess group differences in QoL, whole‐brain vertex‐wise group analyses were performed using FreeSurfer software. Additionally, a general linear model framework, implemented in FreeSurfer, was incorporated. A cluster‐wise correction approach was further applied to address multiple comparisons. A precomputed Z‐Monte Carlo simulation estimated the null distribution of the cluster sizes. Cluster‐forming thresholds were defined at 𝑝 < 0.05, with cluster‐wise significance set at 𝑝 < 0.05. The general linear model was configured to model group differences and covariates of interest, such as age. Contrast matrices were constructed to test the hypotheses of interest, and the results were mapped on the cortical surface for visualization.

### 2.6 Lesion Volumetry by Icobrain MS

3D, T1 MPRAGE, and 3D FLAIR datasets obtained from each patient with MS were analyzed using the “icobrain ms” software by uploading the digital imaging and communications in medicine data files to the Icometrix website (http://icometrix.com) as previously described (Fujimori, Fujihara, et al. 2020a; Fujimori et al. 2021; Fujimori, Uryu, et al., 2020). The analysis revealed whole‐brain, total grey matter, lesion load, and T1 white matter hypointensity volumes.

### 2.7 Statistical Analyses

All statistical analyses except cluster validation were performed using the JMP Pro version 16.2.0 (SAS Institute Inc., NC, USA). Cluster validation was performed using Python version 3.12.0 (Python Software Foundation). The normality of the data distribution was assessed using the Shapiro–Wilk test. Quantitative variables with normal distribution are presented as means with standard deviations (SD), whereas non‐normally distributed variables are reported as medians with interquartile ranges (IQR) or minimum and maximum values, as appropriate. Non‐parametric correlations between the two quantitative variables were evaluated using Spearman's rank correlation coefficient (rho). Numerical variables between disease groups were compared using the Mann–Whitney *U* test or the Steel–Dwass test, whereas categorical variables were compared using the chi‐squared test. Statistical significance was set at *p* < 0.05. Due to the exploratory nature of the study, the threshold of statistical significance was not adjusted for multiple comparisons (Bender and Lange 2001).

## 3 Results

### 3.1 Patient Clinical Profiles

The study included 75 patients with MS. Table 1 summarizes the clinical and radiological characteristics of the cohort. The median total MSNQ and CogEval raw scores were 17 (IQR, 10–26) and 59 (IQR, 51–67), respectively. Median scores of PCS and MCS in SF‐36 were 50.2 (IQR, 38.1–54.8) and 51.8 (IQR, 46.5–56.7), respectively (Table 1).

**TABLE 1:** Clinical and radiological profiles of patients with multiple sclerosis (*n* = 75).

	*n* (%)
Age (years)^†^	44 (38–51)
Sex	
Female	58 (77%)
Male	17 (23%)
Duration (years)^†^	13 (8–19)
EDSS^†^	2 (0–3.5)
Disease course	
RRMS	56 (75%)
PMS	19 (25%)
DMTs	
Interferon	2 (2.6%)
Dimethyl Fumarate	15 (20%)
Fingolimod	15 (20%)
Siponimod	2 (2.6%)
Natrizumab	9 (12%)
Ofatumab	30 (40%)
None	2 (2.6%)
MSNQ total score^†^	17 (10–26)
SF‐36^†^	
2PCS_J	50.2 (38.1–54.8)
2MCS_J	51.8 (46.5–56.7)
CogEval raw score^*^, ^†^	59 (51–67)
Lesion load (mL)^†^	5.51 (3.02–9.79)
T1WM hypointensity volume (mL)^†^	3.85 (2.22–7.64)
Whole‐brain volume (mL)^†^	1486 (1455–1526)
Grey matter volume (mL)^†^	886 (863–918)

* (*n* = 72).

^†^ Median (IQR). Increased scores indicate greater impairment in EDSS and MSNQ, whereas in SF‐36 and CogEval, higher scores indicate better status.

**Abbreviations**: DMT, disease‐modifying therapy; EDSS, Expanded Disability Status Scale; IQR, interquartile range; MCS, mental component summary; MSNQ, Multiple Sclerosis Neuropsychological Questionnaire; PCS, physical component summary; PMS, progressive MS; RRMS, relapsing‐remitting MS; SF‐36, Medical Outcomes Study Short Form‐36 Health Survey; WM, white matter.

### 3.2 Hierarchical Cluster Analysis

**FIGURE 1:**
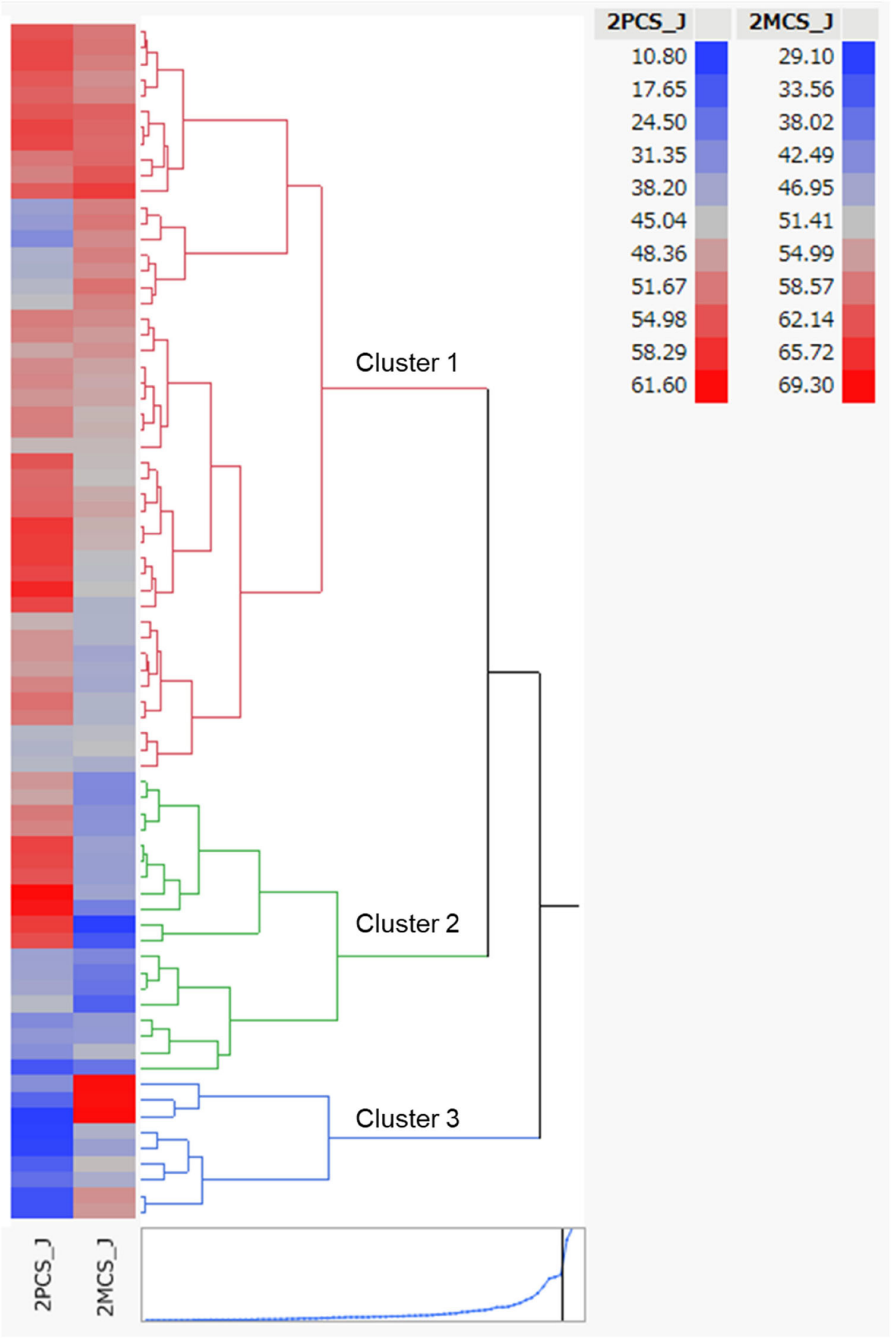
**Hierarchical cluster analysis**. The dendrogram shows hierarchical cluster analysis using two variables—the PCS and MCS—derived from the quality‐of‐life data, classifying patients with MS into three clusters. The distance graph in the lower section shows a noticeable change in slope at three clusters. The change in slope indicates that the differences in clusters that are joined up to the point where three clusters remain are comparatively small. In the dendrogram, the relative distances between clusters are provided by the horizontal distances between the vertical lines that join the clusters. Thus, the distances along the x‐axis represent the similarity between the clusters, with a shorter distance indicating a greater degree of similarity. The vertical axis represents the 75 patients with MS included in the cluster analysis. The color map indicates Cluster 1 (in which PCS and MCS did not decrease), Cluster 2 (displaying decreased MCS), and Cluster 3 (displaying decreased PCS).

The hierarchical cluster analysis results are shown as a dendrogram (Figure 1). The distance graph at the bottom has a noticeable change in slope at three clusters. This change in slope indicates that the differences in clusters that are joined up to the point where three clusters remain are comparatively small. Therefore, we selected the three‐cluster level to categorize patients into subgroups. The values for the silhouette coefficient (0.367), Calinski‐Harabasz Index (52.69), and Davies–Bouldin Index (1.115) supported this selection. Cluster centroids and within‐cluster standard deviation (Supplementary Table eTable 1), and a 2D PCS‐MCS scatter plot showing cluster distribution (Supplementary Figure eFigure 1) are provided. However, because the mean bootstrap Jaccard Index was low, the clustering structure may be sensitive to sampling variability and should be interpreted with caution.

The dendrogram shows how the clustering was conducted. The clustering process can be understood by reading the dendrogram from left to right. Each step consists of the combination of the two clusters that are closest to each other into a single cluster. In the dendrogram, the relative distances between clusters are provided by the horizontal distances between the vertical lines that join the clusters. As patients with MS were classified into three major clusters with long distances, we considered that three clusters corresponding to different QoL subtypes could be defined.

**FIGURE 2:**
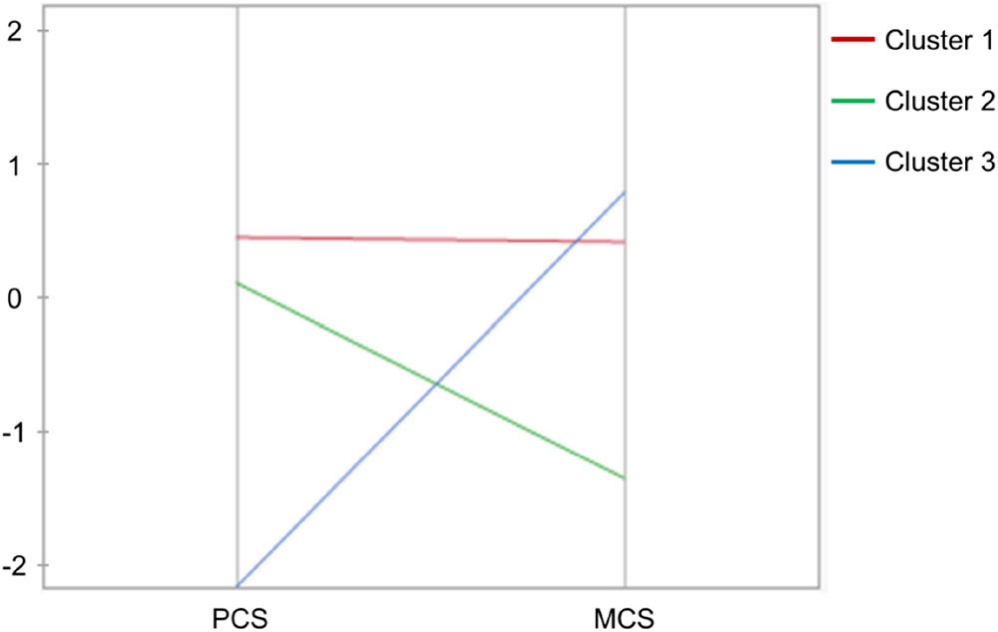
**Cluster means**. Compared to Cluster 1, in which the PCS and MCS derived from the quality‐of‐life data did not decrease, Cluster 2 exhibited a decreased mean MCS, and Cluster 3 exhibited a decreased mean PCS. In the parallel plot of the cluster means, the Y‐axis ranges from two standard deviations above and below the mean, with the SD and mean computed using raw data.

Clusters 1, 2, and 3 comprised 47, 19, and 9 patients with MS, respectively. The mean PCS scores in these clusters were 49.9, 45.8, and 18, respectively, whereas the mean MCS scores were 54.3, 41.5, and 57, respectively. When the three clusters are compared, Cluster 1 showed no reduction in PCS or MCS scores, while Cluster 2 exhibited reduced MCS scores and Cluster 3 had reduced PCS scores (Figures 1 and 2).

### 3.3 Correlation Between PCS and MCS Scores

**FIGURE 3:**
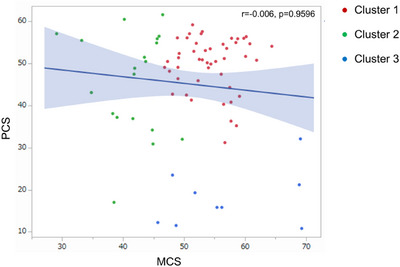
**Scatter plot of the physical component summary and mental component summary of quality‐of‐life data**. The summary scores of the physical and the mental components of QOL did not correlate in patients with multiple sclerosis.

PCS and MCS scores were not correlated. The scatter plot of the PCS and MCS scores indicated that physical and mental QoL did not always correlate in patients with MS (Figure 3).

### 3.4 Comparison Among Three Clusters Regarding Clinical and Icobrain MS Data

Based on the results shown in Figures 1 and 2, we defined Cluster 2 as the cluster associated with reduced mental QoL and Cluster 3 as the cluster associated with reduced physical QoL. Most of the MS cases included in Cluster 1 had higher PCS and MCS scores compared to the average values for healthy individuals (50 in each case), while some cases did not (Supplementary Figure eFigure 1). Therefore, patients in Cluster 1 were considered to have experienced no or mild decline in QoL. Patients with reduced physical QoL (Cluster 3) were older than those in Cluster 1 (*p* = 0.0485) and Cluster 2 (*p* = 0.0364), although these differences were no longer significant after controlling for false discovery rate across non‐imaging tests (Table 2). The proportions of patients with relapsing and progressive MS differed significantly among the three clusters (*p* = 0.0072), with a greater proportion of patients with progressive disease in Cluster 3 (corresponding to reduced physical QoL). Furthermore, patients included in this cluster exhibited significantly higher EDSS scores than those of patients included in Cluster 1 (*p* = 0.0001) and Cluster 2 (*p* = 0.0065). In contrast, patients with reduced mental QoL (Cluster 2) had significantly higher MSNQ scores than those included in Cluster 1 (*p* = 0.001). Analyses of covariance (ANCOVA) were used to compare clinical features among three clusters, with age, sex, and disease duration as covariates. The differences in EDSS, PCS, and MCS were statistically significant, while that in MSNQ was marginal (Supplementary Table eTable 2).

**TABLE 2:** Comparison of clinical data among clusters.

					*p*‐value (FDR‐p)	
	CL1 (*n* = 47)	CL 2 (*n* = 19) (reduced mental QoL)	CL 3 (*n* = 9) (reduced physical QoL)	CL3 vs. CL1	CL3 vs. CL2	CL2 vs. CL1
Age (years)^†^	44 (35–51)	43 (38–50)	51 (45.5–58)	**0.0485** (0.1164)	**0.0364** (0.0971)	0.9911 (0.9997)
Sex (female, male)	38, 9	15, 4	5, 4		0.2472 (0.4238)	
Duration (years)^†^	10 (5–17)	15 (12–22)	13 (9.5–24.5)	0.2659 (0.4245)	0.9997 (0.9997)	0.0645 (0.1407)
RMS, PMS	39, 8	14, 5	3, 6		**0.0072 (0.0216)**	
DMT (base, high, none)	13, 32, 2	4, 15, 0	0, 9, 0		0.3016 (0.4258)	
EDSS^†^	1 (0–2.5)	2 (1–4.5)	6.5 (3.75–7.5)	**0.0001 (0.0008)**	**0.0065 (0.0216)**	0.077 (0.154)
MSNQ^†^	14 (7–21)	25 (18–31)	15 (9.5–39.5)	0.4367 (0.5516)	0.6638 (0.7586)	**0.001 (0.004)**
SF‐36 (2PCS_J) ^†^	51 (46.4–54.8)	48.9 (36.9–55.9)	15.8 (11.9–22.3)	**<0.0001(0.0008)**	**0.0003 (0.0014)**	0.6541 (0.7586)
SF‐36 (2MCS_J) ^†^	54 (50.9–57.7)	41.9 (38.5–45.5)	55.4 (48.4–69)	0.9885 (0.9997)	**0.0002 (0.0012)**	**<0.0001(0.0008)**
	CL1 (n = 47)	CL2 (n = 19)	CL 3 (*n* = 6)	CL3 vs. CL1	CL3 vs. CL2	CL2 vs. CL1
CogEval raw score^†^	63 (54–69)	58 (50–65)	50 (43–58.8)	0.1034 (0.1909)	0.3742 (0.4989)	0.283 (0.4245

^†^ Median (IQR). Bold fonts indicate *p*‐values below 0.05.

**Abbreviations**: Base, baseline therapy (Interferon and Dimethyl Fumarate); CL, cluster; DMT, disease‐modifying therapy; EDSS, Expanded Disability Status Scale; FDR, false discovery rate; High, high efficacy therapy (Fingolimod, Siponimod, Natrizumab, and Ofatumab); IQR, interquartile range; MCS, mental component summary; MSNQ, Multiple Sclerosis Neuropsychological Questionnaire; PCS, physical component summary; PMS, progressive MS; RMS, relapsing MS; SF‐36, Medical Outcomes Study Short Form‐36 Health Survey.

In contrast, there were no significant differences according to sex, disease duration, DMT, or CogEval raw score among the three clusters (Table 2). The number of DMT classes per cluster is provided in Supplementary Table eTable 3. After adjustment for age and education, partial correlation analysis showed that the correlations between MSNQ and CogEval raw score and between MCS and MSNQ were statistically significant, while the correlation between MCS and CogEval raw score was not (Supplementary Table eTable 4).

Whole‐brain volume was significantly decreased in patients with reduced physical QoL (Cluster 3) compared to those in Cluster 1 (*p* = 0.0223). Furthermore, grey matter volume was significantly reduced in these patients compared to those in Cluster 1 (*p* = 0.0101) and Cluster 2 (*p* = 0.042). In contrast, patients with reduced mental QoL (Cluster 2) exhibited a significantly greater lesion load (*p* = 0.0274) and T1 white matter hypointensity volume (*p* = 0.0155) than those in Cluster 1 (Table 3).

**TABLE 3:** Comparison of icobrain MS data among clusters.

					p‐value	
	CL1 (*n* = 47)	CL 2 (*n* = 19) (reduced mental QoL)	CL 3 (*n* = 9) (reduced physical QoL)	CL3 vs. CL1	CL3 vs. CL2	CL2 vs. CL1
Lesion load (mL)^†^	4.27 (1.87–6.46)	8.8 (4.13–12.68)	7.98 (2.38–17.56)	0.5045	0.9182	**0.0274**
T1 WM hypointensity (mL) ^†^	3.11 (1.29–4.66)	6.84 (2.9–8.3)	7.06 (1.86–13.09)	0.4637	0.9672	**0.0155**
Whole‐brain volume (mL) ^†^	1496 (1459–1542)	1485 (1453–1527)	1409 (1378–1493)	**0.0223**	0.1403	0.8525
Grey matter volume (mL) ^†^	897 (868–921)	897 (878–924)	850 (813–889)	**0.0101**	**0.042**	0.9996

^†^Median (IQR). Bold fonts indicate *p*‐values below 0.05.

**Abbreviations**: CL, cluster; IQR, interquartile range; WM, white matter.

### 3.5 Comparison of Cortical Thickness After Adjusting For Age Among The Three Clusters

**FIGURE 4:**
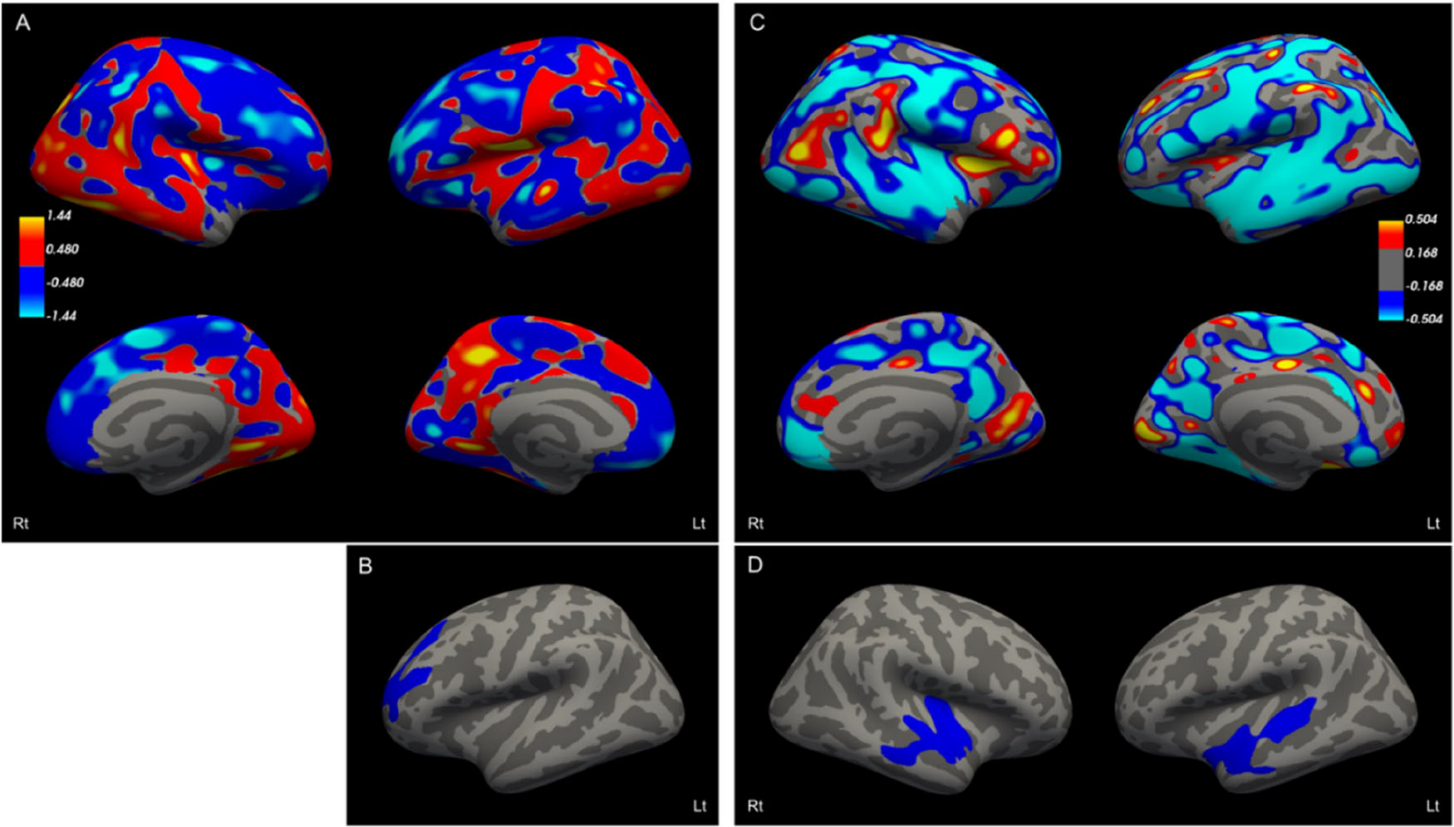
**Different patterns of cortical atrophy**. Cortical atrophy in patients with multiple sclerosis with predominant decrease in physical **(A, B)** and mental **(C, D)** QoL. Patients in Cluster 3 (reduced physical QoL) exhibited decreased cortical thickness in the frontal lobes bilaterally (A, blue areas), with the left rostral middle frontal cortex showing a significant decrease compared to patients in Cluster 1 (B). Patients in Cluster 2 (reduced mental QoL) exhibited decreased cortical thickness in the temporal lobes bilaterally (C, blue areas), with the right insula and left superior temporal cortex showing a significant decrease (D). Lateral and medial‐side images (A, C) and lateral‐side images (B, D) are shown.

Patients with reduced physical QoL (Cluster 3) exhibited decreased cortical thickness in the frontal lobes bilaterally, with a significant decrease in the left rostral middle frontal cortex compared to patients in Cluster 1 (Figures 4A and 4B). In contrast, patients with reduced mental QoL (Cluster 2) exhibited decreased cortical thickness in the temporal lobes, also bilaterally, with a significant decrease in the right insula and left superior temporal cortex (Figures 4C and 4D). However, when we adjusted the comparison for age, sex, disease duration, and TIV, we could not detect any significant differences.

## 4 Discussion

### 4.1 Research Summary

The present study found no significant correlation between reduced physical and mental QoL in patients with MS. Physical or mental QoL decreased in a fraction of patients with MS. Those with a diminished physical QoL were generally older, more likely to have a progressive disease course, and had higher EDSS scores. Neuroimaging displayed reduced whole‐brain and grey matter volumes, along with decreased cortical thickness in the frontal lobes bilaterally, particularly in the left rostral middle frontal cortex. Conversely, patients with reduced mental QoL were more likely to present a relapsing‐remitting disease course and increased MSNQ scores. These patients exhibited increased lesion load, T1 white‐matter hypointensity volume, and decreased cortical thickness in the temporal lobes bilaterally, in the right insula, and in the left superior temporal cortex.

### 4.2 Clinical and General Radiological Findings Associated With Reduced Physical QoL

We considered the results of patients with MS with a predominant decrease in physical QoL to be clinically meaningful, although the proportion of patients in this subset represents only 12% of the total. These patients had progressive disease courses, more pronounced brain atrophy, and greater physical disability. In addition to older age, higher age at disease onset may also contribute to poor prognosis, as suggested by previous research (Rotstein and Montalban 2019), particularly since disease duration did not significantly differ among the three groups. Although spinal cord evaluation was not conducted in this study, there is a possibility of pyramidal tract disturbance in this group due to motor cortex atrophy.

### 4.3 Clinical and General Radiological Findings Associated With Reduced Mental QoL

Approximately a quarter of the patients with MS exhibited a predominant reduction in their mental QoL. We considered the increased MSNQ scores in these patients to be clinically meaningful for the following reasons: Increased MSNQ scores have been associated with depression (Van Laethem et al. 2022). Moreover, patients with decreased mental QoL tended to have a relapsing‐remitting course, as well as increased lesion load and T1 white‐matter hypointensity volume, suggesting an inflammatory rather than neurodegenerative change. Cognitive impairment and depression have been linked to disconnection (Di Filippo et al. 2018; Martino et al. 2021; van Geest et al. 2019). We hypothesize that the increase in lesion load and T1 white‐matter hypointensity volume observed in our study is compatible with disrupted network connectivity, contributing to the decrease in mental QoL.

### 4.4 Frontal Cortex Atrophy Associated With Reduced Physical QoL

Patients with MS with a predominant decrease in physical QoL exhibited reduced cortical thickness in the frontal lobes bilaterally, particularly in the left rostral middle frontal cortex. The prefrontal cortex can be broadly divided into the dorsolateral prefrontal cortex (DLPC), orbital frontal cortex, and medial prefrontal cortex. The DLPC is visible on the lateral aspect of the hemisphere and consists of the anterior portions of the superior and middle frontal gyri, centered on the Broadmann area 9 and including the adjacent Broadmann areas 10 and 46. This region is essential for the executive functions of the brain, including the ability to plan and sequence activities, focus on tasks, and shift and adapt performance patterns to changing circumstances, as well as for working memory (Swenson and Gulledge 2017). The DLPC can be cytoarchitectonically divided into two or three subregions, but their functional role remains unclear (Jung et al. 2022). As the rostral middle frontal gyrus is a subregion of the DLPC, our results suggest that the decreased physical QoL observed in some patients with MS may be due to atrophy in the cortex, which is associated with executive function. It is necessary to study specific executive functions in these patients to confirm this anatomical connection.

### 4.5 Insula and Superior Temporal Sulcus Atrophy Associated To Reduced Mental QoL

Patients with MS and decreased mental QOL exhibited reduced cortical thickness in the temporal lobes bilaterally, including the right insula and the left superior temporal cortex. The insula integrates interoceptive and exteroceptive information to compute and generate subjective emotional states (Pena‐Casanova et al. 2024). Furthermore, the insula plays a crucial role in the integration of cognitive and motivational dimensions through prefrontal subregions such as the dorsolateral and ventromedial prefrontal cortices. Dysfunction of the insula has been linked to disruption of the emotional, mental, and motivational dimensions in various psychiatric disorders (Namkung et al. 2017). Several recent studies have reported that higher fatigue levels in patients with MS were associated with enhanced atrophy in the temporal lobe, insula (Ziccardi et al. 2022), and basal ganglia (DeLuca 2024; Langley et al. 2023). Patients with MS who exhibited cognitive impairment also showed enhanced atrophy in regions that are crucial for cognitive performance, such as the limbic lobe, cerebellum, and insula, compared to cognitively normal patients (Ziccardi et al. 2022). The same group also evaluated which early predictors of cognitive status were capable of predicting long‐term global cognitive impairment 20 years after MS diagnosis and reported that atrophy of the precuneus, insula, parahippocampal, and cingulate gyri were the best predictors for this (Ziccardi et al. 2024). Furthermore, the association cortex of the superior temporal gyrus is implicated in complex social and linguistic functions (Barger et al. 2015). Socio‐cognitive deficits are common among individuals with MS, and several studies have shown significant correlations between cortical thickness (including in the superior temporal gyrus) and dysfunction of social cognition in patients with MS (Batista et al. 2017; Yokote et al. 2021). Key components of social cognition include the theory of mind (the ability to attribute mental states to oneself and others), emotion recognition (the ability to identify and respond to emotional expressions), empathy (the capacity to understand and share the feelings of others), and social perception (the ability to decode social cues and contexts). These cognitive processes are critical for effective communication and social functioning, and their impairments can significantly affect QoL and social interactions.

### 4.6 Limitations

This study had some limitations. First, it was a cross‐sectional, single‐center study conducted with a limited sample size, which may have introduced some selection bias, and cluster case proportions may vary across the population. Cluster size imbalance, especially for the group of patients exhibiting decreased physical QoL, may affect the generalizability of the findings. Second, the lack of a control group of healthy individuals limits the applicability of the structural differences found in the brains of the participants. Third, the presence of mental conditions that may have affected the cognitive profiles and QoL of patients with MS, such as depression, anxiety, fatigue, or sleep disturbances, was not evaluated. Fourth, this study was exploratory in nature and aimed to generate hypotheses rather than confirm them. Therefore, no multiple comparison correction was applied. The reported *p*‐values should be interpreted with caution, and further confirmatory studies are needed to validate the findings reported. Last, our analysis was based on subjective assessments. However, as no objective method for assessing the mental and physical aspects of QoL is currently available, the SF‐36 was selected as our method of choice.

## 5 Conclusion

In this study, we showed that either physical or mental QoL can be primarily affected in a subset of patients with MS, suggesting that approximately a quarter of them might exhibit a predominant decrease in their mental QoL. Those with decreased physical QoL had a progressive disease course, enhanced brain atrophy, and higher physical disability, whereas those with reduced mental QoL showed changes associated with inflammatory processes and suggestive of disrupted network connectivity. Furthermore, patients with MS with poor physical and mental QoL showed cortical atrophy in the precentral anterior and lateral postcentral regions, respectively. Identifying these clinical and radiological findings may help distinguish patients with MS in whom mental QoL is predominantly affected. This, in turn, suggests the need to monitor the mental status of patients to identify mental QoL decline from early on and prevent the so‐called “smouldering‐associated worsening” (Scalfari et al. 2024). Further research on the connections of the cerebral cortex, including the pyramidal tracts, sensory tracts, and the limbic system, may deepen our understanding of this issue.

## Author Contributions

**Juichi Fujimori**: writing – review and editing, writing – original draft, visualization, project administration, methodology, investigation, formal analysis, data curation, conceptualization. **Michiko Nei**: data curation. **Shu Umezawa**: visualization, investigation, formal analysis. **Tatsuro Misu**: supervision. **Ichiro Nakashima**: data curation, resources.

## Funding

This work was supported by JSPS KAKENHI [grant number 24K10665] and the MHLW program [grant number 20FC1030]. IN received research support from LSI Medience.

## Ethics Statement

The study protocol was approved by the Institutional Ethics Committee of the Tohoku Medical and Pharmaceutical University (2023‐2‐002).

## Consent

Written informed consent was obtained from all the participants.

## Conflicts of Interest

JF has received speaker honoraria from Novartis Pharma, Biogen Japan, Chugai Pharma, Takeda Pharmaceutical, and Eisai; and grants from the Japan Society for the Promotion of Science and the Japanese Ministry of Health, Labour, and Welfare Program. IN serves on the scientific advisory boards of Biogen Japan and Novartis Pharma and receives honoraria for speaking engagements with Biogen Japan, Mitsubishi Tanabe Pharma, Novartis Pharma, Takeda Pharmaceutical, and Eisai. TM received speaker honoraria from Tanabe Mitsubishi Pharma, Chugai Pharma, Novartis Pharma, Alexion Pharma, Teijin Pharma, Viela Bio, and Biogen Idec Japan and received research support from Cosmic Corporation and Medical and Biological Laboratories Co. and received a Grant‐in‐Aid for scientific research from the Ministry of Education, Culture, Sports, Science, and Technology. MN and SU have no conflicts of interest to disclose.

## Supporting information




**Supplementary Figures**: brb371050‐sup‐0001‐Figure.docx

**Supplementary Tables**: brb371050‐sup‐0002‐Table.docx

## Data Availability

Individual‐level data cannot be made publicly available due to legal restrictions. However, some anonymised data supporting the findings of this study will be made available upon reasonable request to the corresponding author.
