# Supplementary material for: Differences in the Neural Substrate for Physical and Mental Quality of Life in Patients With Multiple Sclerosis
Source: Brain Behav. 2025 Nov 21;15(11):e71050. doi: 10.1002/brb3.71050 (PMC12638440; doi:10.1002/brb3.71050)
Supplement: Supplementary file 1 — Supplementary Figures: brb371050‐sup‐0001‐Figure.docx [file BRB3-15-e71050-s001.docx]

eFigure 1. 2D PCS-MCS scatter plot showing the cluster distribution.
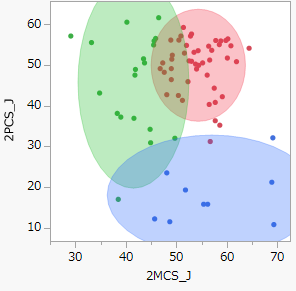


The scatter plot shows the physical component summary (PCS) and mental component summary (MCS) of the quality-of-life data. The red, green, and blue dots indicate clusters 1, 2, and 3, respectively.
