# Supplementary material for: Differences in the Neural Substrate for Physical and Mental Quality of Life in Patients With Multiple Sclerosis
Source: Brain Behav. 2025 Nov 21;15(11):e71050. doi: 10.1002/brb3.71050 (PMC12638440; doi:10.1002/brb3.71050)
Supplement: Supplementary file 2 — Supplementary Tables: brb371050‐sup‐0002‐Table.docx [file BRB3-15-e71050-s002.docx]

eTable 1. Cluster centroids and within-cluster standard deviation.

| Cluster | PCS | MCS |
| --- | --- | --- |
| 1 | 49.9 ± 6.41 | 54.3±4.35 |
| 2 | 45.8 ± 12.1 | 41.5± 5.11 |
| 3 | 18.0 ±6.88 | 57.0 ± 9.65 |

PCS, physical component summary; MCS, mental component summary.

eTable 2. ANCOVA of clinical data between the three clusters adjusted for age, sex, and disease duration.

|  | DF | Sum of Squares | F Ratio | p value |
| --- | --- | --- | --- | --- |
| EDSS | 2 | 52.455986 | 8.2323 | **0.0007** |
| MSNQ | 2 | 686.79831 | 3.104 | 0.0521 |
| 2PCS_J | 2 | 2563.4484 | 20.6699 | **<0.0001** |
| 2MCS_J | 2 | 1238.5056 | 20.0903 | **<0.0001** |

Bold fonts indicate p-values below 0.05. ANCOVA, Analyses of covariance; DF, degrees of freedom; EDSS, Expanded Disability Status Scale; MSNQ, Multiple Sclerosis Neuropsychological Questionnaire; PCS, physical component summary; MCS, mental component summary.

eTable 3. DMTs among clusters.

|  | Cluster 1 (n=47) | Cluster 2 (n=19) | Cluster 3 (n=9) |
| --- | --- | --- | --- |
| Baseline | n=13 (28%) | n=4 (21%) | n=0 (0%) |
| High Efficacy | n=32 (68%) | n=15 (79%) | n=9 (100%) |
| None | n=2 (4%) | n=0 (0%) | n=0 (0%) |

DMT, disease-modifying therapy; Base, baseline therapy (Interferon and Dimethyl Fumarate); High, high efficacy therapy (Fingolimod, Siponimod, Natrizumab, and Ofatumab).

eTable 4. Partial correlation coefficients among neuropsychological scores.

|  | CogEval | MSNQ | 2MCS_J | Age | Education level |
| --- | --- | --- | --- | --- | --- |
| CogEval | . | r=-0.3119  (**p=0.0077**) | r=-0.1479 (p=0.2150) | r=-0.2265 (p=0.0557) | r=0.1031 (p=0.3887) |
| MSNQ | r=-0.3119 (**p=0.0077**) | . | r=-0.3995 (**p=0.0005**) | r=0.1437 (p=0.2284) | r=-0.1880 (p=0.1139) |
| 2MCS_J | r=-0.1479 (p=0.2150) | r=-0.3995 (**p=0.0005**) | . | r=0.1022 (p=0.3931) | r=0.0118 (p=0.9219) |
| Age | r=-0.2265  (p=0.0557) | r=0.1437 (p=0.2284) | r=0.1022 (p=0.3931) | . | r=-0.1084 (p=0.3648) |
| Education level | r=0.1031  (p=0.3887) | r=-0.1880 (p=0.1139) | r=0.0118 (p=0.9219) | r=-0.1084 (p=0.3648) | . |

The values in parentheses indicate the p-values. Bold fonts indicate p-values below 0.05. CogEval, Raw score of CogEval.
